# Toll-like Receptors as Pro-Thrombotic Drivers in Viral Infections: A Narrative Review

**DOI:** 10.3390/cells12141865

**Published:** 2023-07-16

**Authors:** Benjamin Panzer, Christoph W. Kopp, Christoph Neumayer, Renate Koppensteiner, Alicja Jozkowicz, Michael Poledniczek, Thomas Gremmel, Bernd Jilma, Patricia P. Wadowski

**Affiliations:** 1 Department of Cardiology, Wilhelminenspital, 1090 Vienna, Austria; benjamin.s.panzer@gmail.com; 2 Division of Angiology, Department of Internal Medicine II, Medical University of Vienna, 1090 Vienna, Austria; christoph.kopp@meduniwien.ac.at (C.W.K.); renate.koppensteiner@meduniwien.ac.at (R.K.); 3 Division of Vascular Surgery, Department of Surgery, Medical University of Vienna, 1090 Vienna, Austria; christoph.neumayer@meduniwien.ac.at; 4 Faculty of Biophysics, Biochemistry and Biotechnology, Department of Medical Biotechnology, Jagiellonian University, 30-387 Krakow, Poland; alicja.jozkowicz@uj.edu.pl; 5 Division of Cardiology, Department of Internal Medicine II, Medical University of Vienna, 1090 Vienna, Austria; michael.poledniczek@meduniwien.ac.at; 6 Institute of Cardiovascular Pharmacotherapy and Interventional Cardiology, Karl Landsteiner Society, 3100 St. Pölten, Austria; thomas.gremmel@mistelbach.lknoe.at; 7 Department of Internal Medicine I, Cardiology and Intensive Care Medicine, Landesklinikum Mistelbach-Gänserndorf, 2130 Mistelbach, Austria; 8 Department of Clinical Pharmacology, Medical University of Vienna, 1090 Vienna, Austria; bernd.jilma@meduniwien.ac.at

**Keywords:** toll-like receptors, glycocalyx, SARS-CoV-2 infection, platelet activation, micro-thrombosis

## Abstract

Toll-like receptors (TLRs) have a critical role in the pathogenesis and disease course of viral infections. The induced pro-inflammatory responses result in the disturbance of the endovascular surface layer and impair vascular homeostasis. The injury of the vessel wall further promotes pro-thrombotic and pro-coagulatory processes, eventually leading to micro-vessel plugging and tissue necrosis. Moreover, TLRs have a direct role in the sensing of viruses and platelet activation. TLR-mediated upregulation of von Willebrand factor release and neutrophil, as well as macrophage extra-cellular trap formation, further contribute to (micro-) thrombotic processes during inflammation. The following review focuses on TLR signaling pathways of TLRs expressed in humans provoking pro-thrombotic responses, which determine patient outcome during viral infections, especially in those with cardiovascular diseases.

## 1. Toll-like Receptors’ Role in Inflammation and Thrombosis

Toll-like receptors (TLRs) play a major role in the modulation and progression of inflammation as a result of different pathogens such as bacteria, fungi and viruses [1,2]. Once they are synthesized in the endoplasmic reticulum, TLRs are transported to endosomal or plasma membranes [3]. TLRs mediate, as well as propagate, inflammation and, depending on how strongly a TLR is activated, the initiated responses can be beneficial or harmful to the host [4].

Pro-inflammatory diseases increase the rates of thrombo (-embolic) events as a result of increased thrombin generation due to inflammation [5]. Thrombin is the strongest human platelet activator [6] and systemic inflammation plays a major role in atherosclerosis [7]; hence, TLRs also have a direct effect on platelets’ capacity to modulate inflammation [8,9]. Pathways of immuno-thrombosis, initiated by platelet activation following viral sensing, are crucial in the pathogenesis of viral infections [10,11,12]. Herein, TLR-3, TLR-9 and TLR-10 respond to double-stranded (ds) ribonucleic acid (RNA), TLR-7 and -8 recognize single-stranded (ss) RNA and TLR-2 and TLR-4 sense viral envelope glycoproteins [13,14]. However, other TLRs also play a role in viral recognition [13,15]. TLR-3 has been shown to be involved in the development of the immune response to severe acute respiratory syndrome coronavirus type 2 (SARS-CoV-2) and Middle East respiratory syndrome coronavirus (MERS-CoV) [16]. Increased levels of RNA transcription similar to the profile of TLR-3 were observed on the second day after coronavirus infection, shown in a model of non-sterile inflammation induced by viral injection in mice [16].

This increase in TLR-3 activity enhances downstream activities of toll-interleukin receptor (TIR) domain-containing adaptor protein inducing interferon (TRIF), interferon regulatory factor 3 (IRF3), NF-kB and pro-inflammatory cytokines, all of which can induce pro-coagulatory and pro-thrombotic responses [16]. During viral or bacterial infection type I interferon (IFN), signaling is induced upon detection of pathogens (by pathogen-associated molecular patterns) via pattern recognition receptors (PRRs) [17]. IRF3 is activated immediately after viral infection occurs and is a primary activator of IFN genes and the RANTES chemokine gene, finally resulting in the recruitment of leukocytes to sites of inflammation [18]. IFN signaling induces coagulation by activating the coagulation cascade by increasing the expression of high mobility group box 1 (HMGB1), which is a damage-associated molecular pattern (DAMP), in the bloodstream. In thrombi, HMGB1 is most commonly expressed on platelets [19]. TRIF redirects HMGB1 to activate granulocytes, monocytes, macrophages and dendritic cells, inducing coagulation [20]. Monocytes react to inflammation by expressing tissue factor, which induces the coagulation cascade. NF-kB further increases inflammation by inducing adhesion molecules necessary for leukocyte binding and transmigration [21].

Contrarily, TLR-3 deficient mice had an increased survival when compared with wild-type mice when subjected to influenza A virus by non-sterile inflammation due to intra-nasal inoculation [22]. Unsurprisingly, the TLR-3 deficient mice had higher viral production in their lungs [22]. Mouse models showed that protease-activated receptor (PAR)-2 suppressed TLR-3 signaling and thus contributed to viral infectivity [23]. These findings were contrary to previous reports showing increased susceptibility to non-sterile influenza A virus infection in PAR-2 deficient mice when compared with wild-type mice [24].

Traditionally, platelets have been thought to play a role in the amplification of the coagulation cascade at the site of vascular injury [25]. More recently, platelets have been recognized as drivers of leucocyte-mediated immunity [26,27]. Platelets express Fc gamma receptor IIa, which increases platelet functions, causing them to form platelet–leukocyte aggregates [28]. These aggregates can trap and immobilize pathogens [29]. Platelets can also actively regulate immuno-thrombosis in various diseases such as infection [30], injury as a result of ischemia and/or reperfusion [31], cardiovascular diseases (CVD) [32], sepsis [33] and cancer [34].

TLRs are central to immuno-thrombotic platelet function via expression on or within immune cells (such as platelets, monocytes/macrophages, neutrophil granulocytes, dendritic cells, natural killer cells and cells of adaptive immunity (T- and B- cells)) and thus play a vital role in the first line of protection from injury and infection [9,35,36,37].

TLRs are among the PRRs [38] and can recognize both PAMPs and DAMPs [39]. PAMPs are derived from pathogens [40], while DAMPs are associated with tissue damage, which is endogenous [38]. As such, TLRs are partially responsible for the elimination of viruses [41]. However, it must be noted, that this positive aspect can also have a negative impact on the host as a result of tissue destruction and persistent inflammation, such as in the pathogenesis of coronavirus disease 2019 (COVID-19) [42].

## 2. TLR Signaling

In humans, 10 types of TLR have been identified that fall into one of two groups based on their expression on different immune cells. The localization of the TLR expression determines the PAMP/DAMP specificity of the TLR [43]. TLRs 3, 7, 8 and 9 are expressed primarily intra-cellularly [44,45], while TLRs 1, 2, 4, 5, 6 and 10 are expressed primarily on the surface of the cell membrane [2,46,47,48]. The site of expression corresponds to their roles [43]. TLRs generally signal by dimerization after ligand binding [49]. The exception is TLR-2, which hetero-dimerizes with TLR-1 or TLR-6 and recognizes tri-acylated (TLR-1) and di-acylated (TLR-6) lipopeptides, respectively [50]. The occurrence of all 10 human TLRs has been described for human platelets [12].

TLRs consist of an extra-cellular leucine-rich repeat (LRR) domain responsible for PAMP and DAMP sensing, a transmembrane helix and a TIR domain located in the cytosol [51]. Downstream signaling involves the adaptor proteins called toll-interleukin-1 receptor resistance (TIR) domain-containing proteins. In humans, these are myeloid differentiation primary response protein 88 (MyD88), MyD88 adaptor-like (MAL) (also called TIR domain-containing adaptor protein (TIRAP)), TRIF (also known as TICAM1) and TRIF-related adaptor molecule (TRAM, also known as TICAM2) and TIR domain sterile alpha and HEAT/Armadillo motif (SARM) [52,53,54]. In contrast to MyD88, MAL, TRIF and TRAM (which have activating functions), SARM has negative regulatory effects on TRIF-dependent signaling [52,53,54,55]. Importantly, various TLRs use different TIR domain-containing adaptor proteins and induce a broad set of intra-cellular signal transduction pathways, which predominantly result in NF-κB activation [54].

In addition, a pro-apoptotic PI3K/AKT/GSK-3β pathway has been described in rats. [56,57].

TLRs initiate immune responses by activating transcription factors of the nuclear factor-κB (NF-κB) and the interferon regulatory factor (IRF) family in both a MyD88-dependent as well as a MyD88-independent manner [54,58,59]. NF-κB causes the production of pro-inflammatory cytokines and chemokines while also participating in inflammasome regulation. It is also critical in regulating inflammatory T cells and innate immune cells [58]. Modulation of TLR-induced pathways in a positive or negative manner can occur via activation of PI3K by different TLR-agonists such as LPS, CpG, flagellin and by-products resulting from viral infection [60]. In humans, all TLR-receptors utilize MyD88-induced signaling to induce inflammatory cytokine production [61,62]. The impact of MyD88 signaling after TLR-4 activation on inflammatory pathways can also be shown for TLR-3, which was initially thought to exclusively use the TRIF pathway [63]. Once these adaptor proteins bind to TLRs, cytosolic signaling complexes are activated. These contain tumor necrosis associated factor (TRAF) and interleukin receptor associated kinase (IRAK) proteins, which activate NF-κB and IRF, a transcription factor. These in turn trigger the production of pro-inflammatory cytokines and type 1 interferons [54,59]. NF-κB is required for interleukin (IL)-6 and tumor necrosis factor (TNF) production, which in turn activates the transcription of NF-κB [61,64]. As a result of this pro-inflammatory cascade, TLR-1/2 and TLR-4 cause increased P-selectin expression on platelets, activation of integrin alpha(IIb)beta(3) and increased production of reactive oxygen species (ROS) [65,66]. Moreover, thrombin generation can be induced by platelet activation via TLR-2 and TLR-4 [67]. Thus, TLRs can be considered to be the drivers behind the activation of pro-inflammatory processes in platelets, as NF-κB is responsible for first procaspase activating compound (PAC-1) and fibrinogen binding as well as adenosine triphosphate (ATP) release as a result of inflammatory and pro-thrombotic stimuli [68].

## 3. TLRs and Diseases

Viruses, such as severe acute respiratory syndrome corona virus type 2 (SARS-CoV-2), are pro-inflammatory and pro-thrombotic in nature [69]. The cytokine storm induced by SARS-CoV-2 is thought to highly involve TLR signaling [70,71]. Herein, SARS-CoV-2 shares these patho-mechanisms with other viruses; however, distinct differences in the regulation of inflammation and viral persistence can be observed [10,72,73,74]. In addition, SARS-CoV-2 leads to an upregulation of a plethora of TLRs [42,72]. Systemic hyper-inflammation is triggered via the previously outlined mechanism via TLR-2, -4, -6, -7 and -8 [42,72,75]. IL-1β is produced as a result of inflammasome activation and induces IL-6 [76]. High levels of inflammasome activation have been associated with poor outcomes in COVID-19 patients [77]. Moreover, long-lasting inflammatory processes maintaining endothelial dysfunction due to viral persistence might be the underlying cause of thromboembolic events and cardiovascular complications frequently observed in patients suffering from COVID-19 [10,78]. TLR-4 and Nox-2 inhibition is suggested to reduce oxidative stress and platelet-dependent thrombus growth in ex vivo models using the blood of SARS-CoV-2 patients. [79] Moreover, TNF α inhibition also reduces Nox-2-related oxidative stress and platelet activation enhanced by plasma of SARS-CoV-2 patients, thus eliciting signaling pathways in which TLR-4 activation promotes platelet-dependent thrombus growth [79].

There is increasing evidence that TLRs contribute to inflammatory vascular diseases, such as aneurysm formation and different forms of vasculitis [80], and might also be linked to micro- and macro-vascular complications in type 2 diabetes [81,82]. Disease involvement of TLRs is displayed in Table 1.

### 3.1. TLR-1

TLR-1 signaling involves downstream pathways of MyD88 [54]. Hally et al. showed TLR-1 to be significantly upregulated in platelets of patients with acute myocardial infarction (AMI). Platelets from AMI patients and healthy controls were analyzed and compared via Western blotting. While the role of TLR-1 is poorly characterized in AMI patients, it is likely that, similar to the manner in which TLRs exacerbate inflammation, TLR-1 may increase platelet reactivity and therefore thrombosis during and after AMI and thus present a further method of platelet activation [83].

Furthermore, there is evidence that overstimulation of TLR-1 is involved in the pathogenesis of autoimmune diseases such as diabetes mellitus type 1 (DM1) [84], which in itself induces a pro-thrombotic state [85]. The TLR 1/2 pathway, together with TLR-3-induced signaling, is implicated in the defense against chikungunya virus (CHIKV) infection [15]. CHIKV belongs to the alphavirus genus of Togaviridae and is transmitted by female mosquito *Aedes arthropods* [86]. The signaling pathways induced by CHIKV sensing involve MyD88 (by TLR 1/2) and NF-κB, as well as TRIF (by TLR-3) and IRF1 [15]. These pathways result in high IL-27 expression [15]. The latter has pleiotropic effects regarding immuno-modulation and may have implications for the pathogenesis of immune thrombocytopenia [87].

### 3.2. TLR-2

TLR-2 signaling involves downstream pathways of MyD88, TIRAP, TRAM and TRIF [88,89,90].

In addition, SARM is capable of TLR-2 signaling modulation [91].

After dimerization with TLR-1, TLR-2/1 causes platelet activation in a dose-dependent manner when subjected to the TLR-2/1 agonist Pam3CSK4 in both healthy subjects’ and AMI platelets and with and without dual anti-platelet therapy (DAPT, in this case aspirin and clopidogrel or ticagrelor) in in vitro experiments. It has been theorized that, by this mechanism, TLR-2/1 may be involved in the pathogenesis of AMI and could aggravate myocardial ischemia or reperfusion injury and recurrent atherothrombotic events [83].

In the presence of histones, which are released into the circulation during neutrophil extra-cellular trap (NET) formation, thrombin generation is driven by TLR-2- and TLR-4-induced platelet signaling [67]. This enhances platelet activation and promotes further platelet–leukocyte aggregate formation and activation of neutrophils leading to NETosis [10].

Thrombin is the strongest platelet activator and, despite current guideline-driven antiplatelet therapy, thrombin-induced platelet activation still accounts for a considerable and stable platelet aggregate formation [92,93,94].

TLR-2 is also upregulated in patients with abdominal aortic aneurysm (AAA) when compared with healthy individuals; it is currently believed that TLR-2 may be integral at regulating inflammation in the aorta in the context of AAA formation [95].

There is strong evidence that TLRs have a crucial role in the formation of AAA, which are defined as saccular distensions of the abdominal aorta exceeding 30 mm in diameter or 1.5-fold of the regular diameter [96,97,98,99]. The pathogenesis of AAAs is characterized by excessive diapedesis of leukocytes [100], inflammation [101] and the subsequent release of matrix metalloproteinases and elastase from macrophages and lymphocytes that degrade the extra-cellular matrix [102,103] and weaken the vessel wall. While the exact mechanisms that initiate the inflammatory response in the aortic wall have not yet been thoroughly understood, there are hints that TLR activation may be crucial in initiating inflammatory processes, which ultimately lead to AAA formation and atherogenesis [104,105].

There are several studies that have linked TLR-2 and TLR-4 to the formation of AAAs. Yan et al. showed that increased levels of TLR-2 expression were found in human samples of AAA tissue [106]. In addition, the inhibition of TLR-2 in a murine AAA model resulted in a significant reduction in AAA size and TLR-2-deficient mice failed to develop AAAs [106]. Proteins involved in inflammatory downstream signaling pathways, including matrix metalloproteinase and NF-κB, and macrophage recruitment were also significantly reduced in TLR-2-deficient mice [106]. In a TLR-4-deficient murine model of AAA formation, reduced levels of chemokines and interleukins were observed in comparison with a TLR-4-non-deficient murine control group [107].

The role of TLRs for AAA formation was further endorsed by Jabłońska et al. [99], who examined the levels of TLR messenger ribonucleic acid (mRNA) in the blood of AAA patients, healthy volunteers and AAA tissue samples. In the blood, both TLR-2 and TLR-4 mRNA expression was increased in AAA patients compared with control subjects. However, elevated protein levels in serum could only be proven for TLR-4. Compared with the serum levels, TLR-2 expression was increased 20-fold in the AAA specimens [99]. Furthermore, certain polymorphisms in the gene encoding for TLR-2 and TLR-3 were demonstrated to codetermine the risk of AAA formation [98].

Lastly, the knock-out of MyD88, a downstream signaling molecule involved in both the TLR-2 and TLR-4 pathway, reduced both AAA formation and atherosclerosis after angiotensin II infusion in mice predisposed to both disease entities by the knock-out of either apolipoprotein E or low-density lipoprotein receptor (LDL-R) [108]. While in TLR-2 and LDL-R-deficient mice, angiotensin II infusion resulted in AAA formation but not atherosclerosis, both were attenuated in mice deficient in TLR-4 and LDL-R [108].

These findings demonstrate that TLRs and their pro-inflammatory downstream signaling pathways have a crucial role in AAA initiation and formation.

As TLR involvement is crucial in AAA formation, it is not surprising that viral infections such as cytomegalovirus [109] or human immunodeficiency virus (HIV) [110] are discussed to contribute to aneurysm pathophysiology. However, the exact mechanisms and potential novel therapeutic target molecules will need to be identified in future studies.

In viral infections, TLR-2 not only recognizes SARS-COV-2 but is also responsible for sensing the CMV envelope glycoproteins B and H and responding to varicella zoster, vaccinia, Epstein–Barr, hepatitis B and hepatitis C viruses [13,111,112].

Furthermore, Sepehri et al. discussed the upregulated expression of TLR-2 being associated with an increased risk of type 2 diabetes mellitus (DM2). They concluded that, as a result of TLR-2 involvement in activating the innate immune response upon recognition of DAMPs, TLR-2 was responsible for the induction of ROS and inflammatory cytokines, which contributed to the exacerbation of DM2. TLR-2 expression increased in obese patients and correlated with increased serum levels of glucose and free fatty acids. Infections may be considered crucial for the development of DM2 as a result of PAMP-activated TLR-2-initiated pathways and that insulin suppresses TLR-2 expression. These mechanisms shed light on the circulus vitiosus of DM2 [113].

Finally, TLR-2 was shown to have similar involvement in the pathogenesis of auto-immune diseases as TLR-1 (DM1 [84], Graves’ Disease (GD) [114]).

### 3.3. TLR-3

TLR-3 can be stimulated in endothelial cells by endogenous RNA, which is released as a result of apoptosis and necrosis and causes a pro-inflammatory cellular response [115]. Short, single and double strands of RNA result in an inhibition to neo-angiogenesis [116]. TLR-3 signaling is mediated by TRIF and MyD88, hereby conferring the pro-inflammatory responses [63]. Signaling results in the phosphorylation of Akt, ERK1/2 and p38 MAPK and of the subunit p65 of NF-κB [117]. Najem et al. used a cell-permeant nucleic acid stain to test whether TLR-3 was involved in inflammatory venous thrombosis. Polyinosine polycytidylic acid (poly:C), a synthetic double-stranded RNA analog and TLR-3 ligand were given to wild-type mice after FeCl_3_ (non-sterile) induced inferior vena cava injury, increasing the size and cellular density of thrombi when compared with TLR-3 knock-out mice. As a result of this stimulation of the TLR-3 in this model of sterile inflammation, an increased production of reactive oxygen species was observed, as well as increased macrophage and neutrophil recruitment in the wild-type mice. These results strongly suggest that TLR-3 stimulation and RNA release, after endothelial injury, are involved in thrombus formation as a result of the pro-inflammatory response, which leads to the recruitment of macrophages and neutrophils to the injury site [118]. TLR-3 seems to play a more promotional role in platelet activation, as opposed to TLR-2 and -4, which react to classic platelet stimulation by thrombin, ADP or arachidonic acid (AA); in in vitro models TLR-3 activation fails to induce platelet aggregation [117]. However, the presence of suboptimal concentrations of AA, ADP and collagen and thrombin TLR-3 activation by synthetic dsRNA analog lead to a platelet aggregation of 60–80%. Thus, TLR-3 may be considered a promoter for platelet activation [117].

In vivo mouse models showed that TLR-3 knock-out mice reduced coagulatory markers when subjected to poly I:C compared with wild-type mice [119]. Thus, the activation of TLR-3 can induce an endothelial pro-coagulatory state, which can influence cellular hemostasis [119]. There is evidence that the activation of protease activated receptor (PAR)-1 in the presence of dsRNA analog induced INF-β expression in murine models that were infected with coxsackievirus B3 (CBV3). However, this induction of INF-β expression was not present when PAR-2 was activated. Thus, it is believed that PAR-2 negatively regulates TLR-3-dependent INF-β expression [23,120,121]. In vitro experiments using mouse models showed that PAR-4 activation increased chemokine expression, while decreasing TLR-3-related NF-κB expression of pro-inflammatory genes [122]. Wild-type mice had lower immune cell numbers, fewer inflammatory mediators in the lung and decreased mortality when compared with PAR-4 knock-out mice [122].

In a cohort of Danish females, the upregulation of TLR-3 was associated with systemic lupus erythematosus (SLE) [123]. The pro-thrombotic state of SLE was, in part, due to the systemic inflammation and increased circulating immune complexes that were modulated by TLRs [124]. Akin to SLE, TLR-3 is believed to be involved in the pathogenesis of DM1 [84].

TLR-3 is essential for anti-viral activity during rhinovirus infection by inducing IL-6, CXCL8 and CCL5 [125]. Moreover, TLR-3 mRNA expression is induced by rhinovirus replication [125]. During infection with the West Nile virus, TLR-3 response accounts for the development of lethal encephalitis [126]. Herein, the breakdown of the blood–brain barrier is mediated by tumor necrosis factor alpha receptor 1 signaling [126]. It may be assumed that inflammation-mediated endothelial dysfunction with glycocalyx disintegration may be crucial in the disruption of the blood–brain barrier [127].

### 3.4. TLR-4

**Figure 1:**
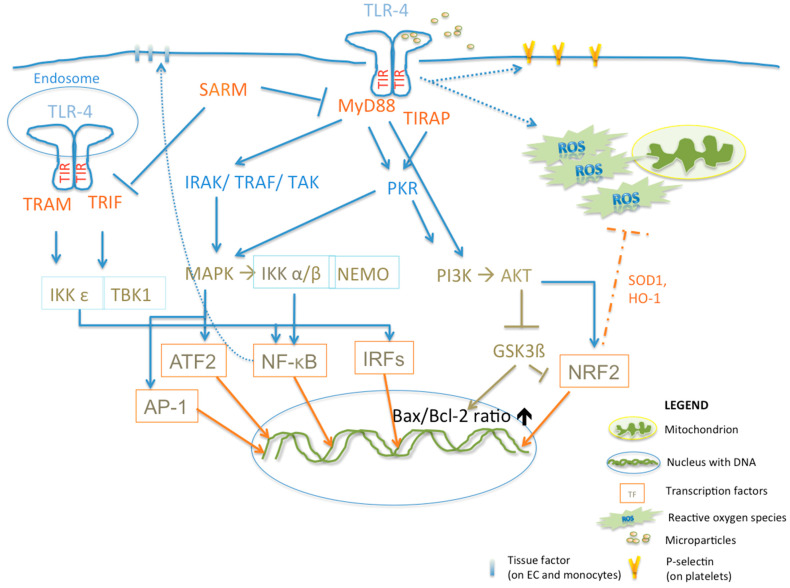
TLR-4-mediated pathways. Abbreviations: AKT—protein kinase B, ATF2—activating transcription factor 2, AP-1—activator protein 1, Bax/Bcl-2 ratio—regulator protein ratio known to be responsible for apoptosis, GSK3ß—glycogen synthase kinase 3 beta, HO-1—heme oxygenase 1, IKKα/β—IκB kinase alpha/beta, IKK ε—IκB kinase epsilon, IRAK—interleukin receptor associated kinase, IRFs—interferon regulator factor, TAK—transforming growth factor-β-activated kinase, TBK1—TANK binding kinase, TIR—toll interleukin receptor, TIRAP—TIR domain-containing adaptor protein, TRAM—TRIF-related adaptor molecule, TRAF—tumor necrosis associated factor, TRIF—TIR domain-containing adaptor protein inducing interferon, MAPK—mitogen-activated protein kinases, MyD88—myeloid differentiation primary response protein 88, NEMO—NF-kappa-B essential modulator/inhibitor of nuclear factor kappa-B kinase subunit gamma, NF-κB—nuclear factor k light chain enhancer of activated B cells, NRF2—nuclear factor erythroid-2-related factor 2, PKR—double-stranded RNA-dependent protein kinase, PI3K—phosphoinositide 3-kinase, ROS—reactive oxygen species, SARM—selective androgen receptor modulators, SOD1—superoxide dismutase 1.

TLR-4 can be activated by various ligands, including lipopolysaccharides, viral glycoproteins, tenascin-C, fibronectin extra domain A and extra-cellular cold-inducible RNA-binding protein (eCIRP) [13,128,129,130,131,132]. The latter is released during sepsis, tissue ischemia–reperfusion injury, trauma and hemorrhage and acts as an endogenous DAMP [132,133]. TLR-4 can activate MyD88- and TIRAP, as well as TRIF-dependent pathways [134,135] (Figure 1).

These pathways, with cross-talks between them, result in the phosphorylation of MAP kinases and activation of IKK alpha/beta, NEMO, IKKε and TBK1, which lead to the phosphorylation and activation of transcription factors such as NF-κB, IRFs, activator protein 1 (AP-1) and activating transcription factor 2 (ATF2) [54,134,136].

Signaling through TIRAP, but also through MyD88, activates double-stranded RNA (dsRNA)-activated protein kinase PKR, which is upstream of MAPK signaling and results in NF-κB activation [135]. PKR has also been shown to be capable of PI3K/Akt pathway activation during neo-vascularization [137]. In addition, the activated PKR signaling might have a modulatory role, as it can also regulate NRF2 activation, a transcription factor promoting the expression of anti-oxidant enzymes such as heme oxygenase 1 (HO-1) or superoxide dismutase 1 (SOD-1) [138,139].

The adaptor TRAM has been described to bridge to TRIF and so both TRAM- and TRIF-associated signaling leads to IRF3 activation via IKKε and TBK1 [54,140]. The fifth adaptor protein, SARM, has been described to negatively regulate MyD88 and TRIF- mediated TLR-4 signaling [52,141].

TLR-4 has also been considered to play a role in the induction of apoptosis and, most prominently, fibrosis [142]. TLR-4 has been suggested to induce apoptosis via the PI3K/AKT/GSK-3β signaling pathway [57].

Significant evidence exists for the participation of TLR-4 in coagulation by several mechanisms [143]. Among these, TLR-4 can promote endothelial and platelet activation; the latter is also mediated by the internalization of micro-particles [144,145].

Moreover, via NF-κB and AP-1 activation, TLR-4 mediates together with TLR-2 tissue factor (TF) expression on endothelial cells [146]. In monocytes, TF expression mediated by TLR-4 and TLR-6 has been described [147].

**Figure 2:**
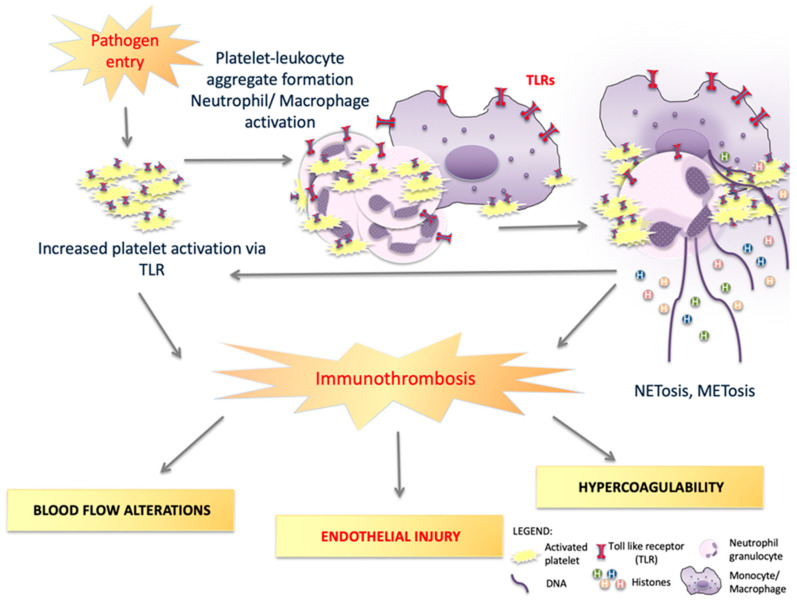
Platelets as drivers of leukocyte-mediated immunity and immuno-thrombosis via toll-like receptors (TLRs).

The dual nature of TLRs on platelets is evident in TLR-4, which has been shown to both augment and inhibit neutrophil responses such as platelet–neutrophil aggregates, neutrophil extra-cellular trap formation and bacterial trapping in septic patients [148]. When a co-culture of neutrophils and platelets is subjected to TLR-4 agonists, CD62L (L-selectin) expression, phagocytosis and IL-8 secretion are increased, while shedding of CD62L and elastase secretion are decreased. Thus, platelet TLR-4 is responsible for neutrophil responses to pathogens and lipopolysaccharides (LPS) [149]. The latter facilitate the aggregation of platelets and neutrophils and the production of NETs [149]. TLR-4 signaling also mediates platelet–monocyte interactions and is required for P-selectin-induced platelet–monocyte aggregation [26,150]. In addition, TLR-4 induces caspase-1 activation and caspase-11 expression, leading to cellular pyroptosis [151]. Caspase-11-mediated inflammatory responses occur partly via gasdermin D-induced pyroptosis in macrophages, a process involved in the pathogenesis of atherosclerosis [152]. Hence, TLR-4 signaling promotes gasdermin D-induced effects. The latter are mediated via the NF-kB pathway [153]. Gasdermin D and caspase-1 signaling have furthermore been shown to be involved in TLR-4-induced macrophage extra-cellular trap (MET) formation and METosis [133]. METosis is a process wherein monocytes or macrophages release anti-microbial proteins and DNA which form extra-cellular traps [133]. Akin to NETosis, METosis leads to the release of DNA, anti-microbial proteins and histones from monocytes or macrophages, promoting extra-cellular trap formation and being a highly potent activator of immuno-thrombosis [133,154] (see Figure 2).

Tissue-type plasminogen activator (tPA) is a major activator of fibrinolysis; the anti-inflammatory properties of enzymatically inactive (EI) tPA are TLR specific. EI tPA, reduces the pro-inflammatory process in bone marrow-derived macrophages (BMDMs) as a result of LPS activity by blocking BMDMs to some of the TLR specific agonists. This inhibits the expression of TNFα and ILs [155]. A deregulated example of this function occurs in diabetics who have wound healing disorders as a result of increased inflammatory activity, in part due to TLRs [156].

There is evidence that TLR-4 may be solely responsible for fibrinous cardiac remodeling after ischemic events. Mouse models have shown that TLR-4 knock-out mice had no evidence of fibrinous remodeling, even in the presence of fibrin modulators and agonists, whereas wild-type mice experienced typical cardiac remodeling with fibrinous elements after permanent ligation of the left descending coronary artery (sham surgery). Furthermore, knock-out TLR-4 mice had reduced left ventricular remodeling and increased preservation in systolic function [157].

Human models have shown significant upregulation of TLR-4 on platelets in patients after experiencing an acute myocardial infarction. Furthermore, in vitro experiments have demonstrated that healthy and AMI platelets are activated in the presence of high doses of the TLR-4 agonist LPS. Increased activation of TLR-4 is also associated with heart failure following AMI [158].

TLR-4 likely plays a key role in the pathogenesis of Graves’ Disease and may contribute to heart failure in these patients [114].

Similar to TLR-2, TLR-4 is upregulated in AAA patients when compared with healthy patients (please see paragraph concerning TLR-2) [95].

During Dengue virus infection, the flavivirus non-structural protein 1 (NS1) mediates TLR-4-associated cytokine production [159]. NS1, which is a secreted glycoprotein from Dengue, Zika, West Nile, Japanese encephalitis and yellow fever viruses, is implicated in viral replication, immune evasion and vascular leakage [160]. Herein, it contributes also to Dengue hemorrhagic fever and shock [42]. The emergence of hyper-permeability, and in turn tissue edema, is induced by the disruption of the glycocalyx components heparan sulfate, sialic acid and syndecan 1 [160,161,162]. This is mediated via the upregulation of the enzymes sialidases and heparanase contributing to glycocalyx degradation [161,163]. However, pathomechanisms seem to be more distinct in Dengue virus infection than in infection with other flaviviruses [163].

Many viruses cause endoplasmic reticular stress when they use the cell machinery to produce large amounts of viral proteins [164]. These stressed or dying cells release TLR-4 agonists, for example, high mobility group protein 1 (HBGP1). This causes an inflammatory reaction, which can also be observed in obese individuals [165].

TLR-4 in neutrophils also has a key role in NET formation, for example, when recognizing respiratory syncytial virus (RSV) fusion protein [166]. In mice, TLR-4 has also been involved in the reactivation of cytomegalovirus, which has been previously intra-peritoneally injected (as a non-sterile infection/inflammatory model), from latency after LPS stimulation [167]. Interestingly, TLR-4 has also had a role in long-term post-COVID-19 sequelae [168,169]. Herein, S100A8/A9, a calcium-binding protein, stimulates the TLR-4/receptor for advance glycation end-products’ (RAGE) pathway and chronically activates IL-1b, IL-6 and tumor necrosis factor (TNF)-alpha expression [168]. Neuro-inflammation triggered by SARS-CoV-2 spike protein, which was injected intra-cerebroventricularly (non-sterile infection model), which binds to TLR-4, has been discussed to mediate long-term cognitive impairment after COVID-19 [169].

### 3.5. TLR-5

TLR-5-mediated signaling involves TIRs, MyD88, TIRAP, TRIF and possibly also TRAM [88].

TLR-5 acts as a sensor for the immune system against bacteria by capturing flagellated bacteria. Flagellin, a structural protein of the flagellum, stimulates inflammatory responses and development of adaptive immunity in humans. Once the protein ligand on the flagellum is bound by TLR-5, MyD88 and TRIF are recruited. This leads to NF-κB activation and cytokine secretion and the inflammatory response is induced [170].

Xiao et al. showed that TLR-5 might be associated with decreased GD susceptibility in female subjects in a Chinese Cantonese population. Gene polymorphisms related to TLR-5 in 332 GD patients compared with 351 healthy controls were associated with a decreased risk of GD in women [171].

TLR-5 expression was significantly elevated in patients with severe COVID-19 [72]. However, TLR-5 has been discussed to confer beneficial effects during viral infections such as influenza A and COVID-19 [80,172]. In influenza A infection, activation of the TLR-5 pathway by flagellin has shown a decrease in viral RNA, possibly independent of signaling via type I interferon and IL-22 [172]. Furthermore, TLR-5 seems to have a role in the inhibition of hepatitis B virus [173]. The involvement of TLR-signaling pathways in hepatitis B pathophysiology is supported by the results of a transgenic mouse model with injections of an anti-CD40 agonist, CD40 alpha, which showed the inhibition of HBV replication by induction of inflammatory cytokines [174].

On the other hand, recent results using non-sterile inflammatory models in mice suggest that, during COVID-19, TLR-5 signaling might enhance SARS-CoV-2 infectivity [175].

### 3.6. TLR-6

TLR-6-mediated signaling involves TIRs, MyD88 and TIRAP [88,135].

TLR-6, along with TLR-4, seems to play a significant role in thrombosis in patients with high levels of LDL. Increased LDL promotes inflammation through oxidative stress. This causes the expression of the pro-coagulatory tissue factor (TF). [176] Owens et al. designed an experiment using animal models, where adding simvastatin reduced the expression of TF. It was further shown that deficiency in TLR-4 and TLR-6 reduced levels of micro-particles in the plasma, reduced expression of TF and reduced coagulation and inflammation in hyper-cholesterolemic mice and monkeys. Thus, the involvement of TLR-6 and -4 may be considered a major contributor to atherosclerosis [147].

The synergistic activation of TLR-2/6 and TLR-9 has been shown to protect mice against non-sterile influenza virus infection in a non-sterile inflammation model in mice [177].

### 3.7. TLR-7 and -8

Signaling through TLR-7 involves TIRs, MyD88, TRAM and also TIRAP [178,179]. Interestingly, TLR-7 and TLR-9 signaling can be modulated by SARM1, which induces via this pathway apoptosis in neurons [180].

TLR-8 mediated signaling involves TIRs, MyD88 and TIRAP [181].

So far, only TLR-7 has been shown to have significant involvement in monocyte conversion to dendritic cells to support the primary immune response against pathogens. This occurs when TLR-7 induces cytokine production in monocytes and disposal of damaged cells. Chronic TLR-7 stimulation causes monocytes to differentiate into macrophages [182].

Moreover, TLR-7 signaling in plasmacytoid dendritic cells (pDCs) involves the translocation of IRF5 and IRF7 from the cytosol to the nucleus and might herein be involved in the activation of pDCs [183].

Both TLRs 7 and 8 bind single-stranded RNA, thus initiating the immune response against viruses [183,184]. Myocardial cells have been shown to express TLR-7 and -8 when subjected to coxsackie B viruses [185]. This may explain the production mechanism of IL-6, INF-β and TNFα in myocarditis patients and, in part, the chronic aspect of the disease [185], while IL-6 promotes platelet production by acting upon megakaryocytes and hepatocytes (increased release of thrombopoietin) [186]. Highly increased levels of IFN-β have been correlated with thrombocytopenia [187]. Finally, TNFα is considered the causal molecule for platelet hyper-reactivity and the formation of larger thrombi in older humans and aged mouse models [188].

Platelets also play a vital role in the immune response to viruses such as influenza virus type A. Platelets express TLR-7 on their cell surface. Once activated, TLR-7 causes platelets to express alpha granules, P-selectin and CD40L, leading to a platelet-driven pro-thrombotic effect. Driven by TLR-7, platelets can engulf the virus, causing the release of complement factor C3, which stimulates the release of neutrophil DNA, thus promoting the formation of platelet–neutrophil aggregates preceding NET formation [189].

TLR-7 has been reported to be involved in type I IFN induction by Middle East respiratory syndrome (MERS) coronavirus (MERS-CoV) [190]. In SARS-CoV-2-infected patients, a decrease in pDCs was observed, correlating with disease severity [191]. It is suggested that SARS-CoV-2 dampens TLR-7 responses through interaction with neuropilin-1 [191]. In pDCs, TLR-7 induced pathways after viral RNA sensing trigger MyD88-IRAK4-TRAF6 signaling, leading to CXCL10 induction as well as IRF7 phosphorylation, translocation mediating type I and III interferon expression [191].

Genetic polymorphisms of TLR-7 and -8 have been shown to predict susceptibility to CHIKV [192]. However, in hepatitis C virus infection, TLR-7, together with TLR-3 signaling, seems to constitute a protective immune response [193]. This hypothesis was corroborated by a study showing that myocarditis (coxsackie B virus) patients with mutant TLR-3 phenotype had increased viral replication when compared with patients with a normal TLR-3 phenotype, thus showing that genetic differences in TLR-3 together with PAR-2 modulation of INF- β effect the host’s vulnerability to viral cardiomyopathies [23,194].

Pro-coagulant pathways have been described after the recognition of HIV nucleic acids by TLR-7 and -8 on neutrophils, leading to NET production [195].

TLR-7 has been shown to be involved in the pathogenesis of SLE in Japanese females, when gene analysis was performed, compared with a healthy control [196], while TLR-8 has been positively correlated to SLE in a Danish population [123].

TLR-7 has been shown to be involved in the pathogenesis of GD [171], while TLR-8 seems to be involved in the pathogenesis of rheumatoid arthritis [197].

Similar to TLR-1, -2 and -3, TLR-7 mediates inflammation contributing to DM1 [84].

### 3.8. TLR-9

TLR-9 is integral to inflammation and metabolism. The pathways induced by TLR-9 activation involve TIRs, MyD88 and TIRAP [198]. In addition, signaling through TRIF has also been shown [199]; studies regarding TRAM are missing [88]. Together with TLR-7, TLR-9 can induce apoptosis via SARM1 [180]. As hitherto known, signaling through TLR-9 can result in NF-κB or IRF-7-dependent type I interferon (IFN) pathway activation [200]. Further, PI3Kγ has also had some critical roles in the modulation of immune responses mediated by TLR-9 [60].

Similar to TLR-7 signaling, TLR-9-mediated signaling in pDCs involves translocation of IRF5 and IRF7 from the cytosol to the nucleus [183].

It is known that, in humans, BAD-LAMP (LAMP5) dampens TLR-9-mediated type I IFN production by control of TLR9 sorting in a different endosome subset [201].

TLR-9 is associated with the pathogenesis of non-alcoholic steatosis hepatis (NASH) and is likely a driver for NASH-associated fibrosis, as it has been shown to be expressed in 13.3% of normal liver tissue, 53.3% in mildly fibrotic patients, 80% in cases of cirrhosis and 95% in hepato-cellular carcinoma patients. TLR-9 is activated by circulating mitochondrial DNA, which is increased in obese individuals, metabolic dysfunction-associated fatty liver disease and NASH [202].

Similar to TLRs 7 and 8, TLR-9 is involved not only in the pathogenesis of auto-immune diseases such as SLE (shown in Asian and Danish cohorts) but also in rheumatoid arthritis and multiple sclerosis [123,203,204].

In mice, TLR-9 is involved in non-sterile cytomegalovirus infection, as shown in a model of non-sterile inflammation [205]. Furthermore, TLR-9 senses herpes simplex virus type 1 and 2 and Epstein–Barr virus [206,207,208,209].

MyD88 activation and signaling through IRAK4 suppresses lytic reactivation of Epstein–Barr virus and favors its latency in B-cells [210]. In peripheral T-cell lymphomas, TLR-9 and programmed cell death-ligand 1 (PD-L1) expression are associated with poor survival [211]. Hence, targeting of both TLR-9 and PD-L1 is suggested to induce a sustained anti-tumor immunity [212].

TLR-9 deficient mice have been compared to wild-type mice with regard to their capability of resolving venous thrombosis. The TLR-9-inhibited and -deficient mice were less capable of resolving venous thrombosis after inferior vena cava ligation when compared with wild-type mice. When wild-type mice were subjected to a TLR-9 stimulant, early venous thrombosis resolution was accelerated [213].

### 3.9. TLR-10

TLR-10 mediated signaling involves MyD88 and possibly also TRIF [214].

TLR-10 seems to be the only human TLR that has an inhibitory function over the innate immune system and inflammation. Its role in this modulatory function within the innate immunity is largely unknown (except an inhibitory effect on TLR-2 responses [215]) and it is assumed that the exact anti-inflammatory properties and the impact on the trained immune response in humans as well as therapeutic options remain to be established [216]. Homo-dimer TLR-10/10 and hetero-dimer TLR-10/2 have been shown to recruit MyD88 [216]. Different ligands are discussed for binding to TLR-10, among those HIV-gp41, in turn promoting IL-8 production and NF-κB activation [217]. Moreover, TLR-10 is able to bind dsRNA in an acidic environment [14]. After recruitment of MyD88, the initiated pathway inhibits the production of interferon regulatory factor-7-dependent type I IFN [14]. In addition, a cross-talk with TLR-3-initiated pathways has been described [14]. Furthermore, Lee et al. have discussed TLR-10 as a relevant viral sensor of innate immunity [218].

## 4. Discussion

The synergistic patho-mechanisms of inflammation and in consequence disturbance of the endothelial surface layer with altered vascular perfusion, (micro-)thrombosis and tissue edema drive life-threatening complications of viral infections [10,219].

TLRs are a key factor in regulating NET formation, as the activation of TLRs on neutrophils triggers NET release and herein the binding, immobilization and inactivation of viruses [220]. Similarly, TLR- 4-induced METosis leads to the release of nuclear and mitochondrial DNA and histones [133]. The released histones are recognized as DAMP and activate platelets via TLR-2 and -4, leading to thrombin generation [67,221] (see also Figure 1). Moreover, signaling through TLR-2 increases vWF release from alpha granules in megakaryocytes/platelets [222,223] and also from Weibel–Palade bodies in endothelial cells [224]. As recently reviewed, these mechanisms are central to SARS-CoV-2 patho-physiology [10] but can also be observed in other viral infections [220,225,226]. One can assume that viral infections leading to (micro-) thrombotic complications are characterized by a TLR-vWF-NETosis axis, which in itself drives the processes of immuno-thrombosis impairing (micro-)vascular integrity. The disturbance of the latter is an underlying cause of Virchow’s triad impairment and drives tissue hypoxia, leading to organ failure [10]. In this context, rheological changes due to infection and inflammation should also be considered [227]. When exposed to oxidative inflammation, red blood cell membrane fluidity decreases, impairing systemic micro-circulation and, therefore, tissue perfusion [228]. The latter could be shown in different cardiovascular diseases [229,230,231,232], where a chronic inflammatory oxidative stress burden co-exists [233,234,235]. Limitation of oxidative injury might be given by PKR activation, which is known to enhance NRF2-mediated gene expression of anti-oxidant proteins such as SOD-1 and HO-1 [138,139].

This could be a negative feedback loop that limits inflammatory processes mediated by TLRs. However, the exact mechanisms should be studied in different conditions of inflammation, e.g., atherosclerosis or ischemia–reperfusion injury and inhibiting concepts such as cellular conditioning or HO-1 induction [236,237,238,239].

It should also be noted that pathways of immuno-thrombosis induced by TLR signaling contribute to changes in the vascular wall, including atherosclerosis and aneurysm development and progress [240,241].

Anemia as a result of inflammatory processes has been previously recognized and widely discussed [242]. Though a result of multiple causes, anemia can also be driven by chronic TLR-7 and TLR-9 signaling, initiating the differentiation of inflammatory hemophagocytes [243]. The latter are also responsible for thrombo-cytopenia [243]. Moreover, infection with SARS-CoV-2 leads to elevated RBC calcium levels, resulting in higher RBC fragility [244]. In hospitalized COVID-19 patients, anemia is linked to decreased survival [245].

Anemia leads to alterations of platelet function with enhanced monocyte–platelet aggregate formation and P-selectin expression, as observed in patients with DAPT consisting of either aspirin and clopidogrel or aspirin and prasugrel/ticagrelor, respectively [246]. Furthermore, the highest risk of ischemic events has been reported in anemic patients with high on-treatment residual platelet reactivity (HRPR); however, the highest risk of bleeding has been reported in anemic patients without HRPR [247].

Therapeutic possibilities influencing TLR pathways are challenging, since they are limited by side effects through pleiotropic functions. In discussion as potential benefits are TLR agonists, such as TLR-3 agonist poly(I:C), which has been shown to confer anti-viral effects in animals [248]. TLR-9 agonism by oligonucleotides enhances cytokine production and modulates viral response [248]. However, it should be noted that the models of TLR agonism represent a “sterile” inflammatory, which might not depict all processes involved after pathogen-induced signaling.

On the other hand, TLR-7/9 antagonists such as chloroquine, hydroxy-chloroquine and quinacrine have been widely used for the treatment of immune-mediated inflammatory disorders (herein, SLE, rheumatoid arthritis, and Sjögren’s syndrome) [249].

The activation of TLR-7 has been suggested to modulate hepatitis B, herpes simplex and human papillomavirus infections [13]. In SARS-CoV-2 infection, TLR-2/6 agonism by INNA-051 has shown promising results in reducing viral RNA levels in a non-sterile infection/inflammation model in ferrets [250]; mouse models have shown that activation of TLRs 2 and 7 induces pro-coagulatory transcription factor expression in (non-sterile) sepsis-induced coagulopathy, making it a possible therapeutic goal in the future [251].

**Table 1:** Diseases linked to toll-like receptors. Table showing an overview of diseases discussed in the manuscript and the TLRs involved in their pathogenesis.

Diseases	TLRs Involved	References
Auto-immune		
Graves’ Disease	1, 2, 5, 7, 8	[114,171]
Multiple Sclerosis	9	[204]
Rheumatoid Arthritis	2, 8, 9	[48,91,197,203]
Systemic Lupus Erythematosus	3, 7, 8, 9	[123,196]
Cardiovascular		
Abdominal Aortic Aneurysm	2, 4	[95,98,99,104,105,106,108]
Acute Myocardial Infarction	1, 2, 4	[83,157,158]
Atherosclerosis	1, 2, 4, 6	[104,152,240]
Vasculitis	4, 5	[80]
Infectious		
Chikungunya Virus	1, 2, 3, 7, 8	[15,192]
Cytomegalovirus	2, 4, 7, 9	[109,167,205]
Coronavirus Disease 2019	2, 4, 5, 6, 7, 8	[42,72,74,75,168,169,175,250]
Dengue Virus	4	[159,160]
Epstein Barr Virus	2, 7, 9	[13,209]
Herpes Simplex Virus 1 and 2	9	[207,208]
Hepatitis B	2, 5, 7	[13,112,173]
Hepatitis C	2, 3, 4, 7	[13,111,193]
Human Immunodeficiency Virus	7, 8, 10	[110,195,217]
Influenza	2, 5, 6, 7, 9, 10	[22,172,177,179,189,218]
Middle Eastern Respiratory Syndrome	3, 7	[16,190]
Respiratory Syncytial Virus	4	[166]
West Nile Virus	3	[126]
Varicella Zoster	2	[13]
Metabolic		
Diabetes Mellitus Type 1	1, 2, 3, 4, 7, 9	[82,84]
Diabetes Mellitus Type 2	1, 2, 4	[81,82,113]
Non-Alcoholic Steatosis Hepatis	9	[202]

In general, our review is intended to raise awareness regarding thrombo-inflammatory pathways mediated by TLR responses. This should give opportunities for hypothesis generation in future research. Although we have used as data source the NCBI database PubMed and the herein indexed publications with a broad strategy on used MESH terms, a limitation to our review is its narrative character, which also mirrors the opinion of the authors.

Moreover, we attempted to describe human TLR receptors and signaling pathways; however, knowledge is often limited by the availability of animal-based models.

## 5. Conclusions

Pathways induced by TLR signaling are complex and can promote beneficial effects such as viral elimination with side effects harming tissue homeostasis [10]. TLR pathways can result in a burst of immuno-thrombosis, resulting in NET and MET production, promoting a pro-inflammatory and pro-thrombotic response destabilizing the equilibrium of vascular and platelet function [148,150]. Moreover, TLR pathways may play an important role in virus reactivation and associated long-term pro-inflammatory responses [167,169].

Patients’ comorbidities and the multi-level effects of viral infections including inflammation-driven pro-thrombotic effects pose therapeutical challenges and the potential for adverse drug interactions without a clear clinical benefit. Further studies to elucidate the cross-talks in TLR signaling with a focus on viral long-term sequelae are warranted.

Pathogen entry causes sensing by TLRs, which in turn activate platelets leading to platelet–leukocyte aggregation [26]. TLR signaling mediates neutrophil and macrophage activation and promotes neutrophil extra-cellular trap (NET) as well as macrophage extra-cellular trap (MET) formation [37,133,150]. NETosis and METosis cause the release of DNA and histones, which, as DAMPs, drive further pro-inflammatory and pro-coagulatory responses via TLRs [133,154]. Immuno-thrombotic processes lead to alterations based on Virchow’s triad, of which endothelial injury with glycocalyx degradation is crucial in patho-physiological processes [10].

## Data Availability

Not applicable.
